# HCV NS5A Up-Regulates COX-2 Expression via IL-8-Mediated Activation of the ERK/JNK MAPK Pathway

**DOI:** 10.1371/journal.pone.0133264

**Published:** 2015-07-31

**Authors:** Wei-Chun Chen, Chin-Kai Tseng, Yen-Hsu Chen, Chun-Kuang Lin, Shih-hsien Hsu, Shen-Nien Wang, Jin-Ching Lee

**Affiliations:** 1 Graduate Institute of Medicine, College of Medicine, Kaohsiung Medical University, Kaohsiung, Taiwan; 2 Institute of Basic Medical Sciences, College of Medicine, National Cheng Kung University, Tainan, Taiwan; 3 Center of Infectious Disease and Signaling Research, College of Medicine, National Cheng Kung University, Tainan, Taiwan; 4 Division of Infectious Diseases, Department of Internal Medicine, Kaohsiung Medical University Hospital, Kaohsiung, Taiwan; 5 School of Medicine, Graduate Institute of Medicine, Sepsis Research Center, Center for Dengue Fever Control and Research, Kaohsiung Medical University, Kaohsiung, Taiwan; 6 Department of Biological Science and Technology, College of Biological Science and Technology, National Chiao Tung University, HsinChu, Taiwan; 7 Doctoral Degree Program in Marine Biotechnology, National Sun Yat-Sen University, Kaohsiung, Taiwan; 8 Division of Hepatobiliary Surgery, Department of Surgery, Kaohsiung Medical University Hospital, Kaohsiung, Taiwan; 9 Department of Surgery, Faculty of Medicine, Kaohsiung Medical University Hospital, Kaohsiung, Taiwan; 10 Department of Biotechnology, College of Life Science, Kaohsiung Medical University, Kaohsiung, Taiwan; 11 Graduate Institute of Natural Products, College of Pharmacy, Kaohsiung Medical University, Kaohsiung, Taiwan; University of Washington, UNITED STATES

## Abstract

Chronic hepatitis C virus (HCV) infection leads to intrahepatic inflammation and liver cell injury, which are considered a risk factor for virus-associated hepatitis, cirrhosis, and hepatocellular carcinoma worldwide. Inflammatory cytokines are critical components of the immune system and influence cellular signaling, and genetic imbalances. In this study, we found that cyclooxygenase-2 (COX-2) and interleukin-8 (IL-8) were significantly induced by HCV infection and HCV NS5A expression, and induction of COX-2 correlated with HCV-induced IL-8 production. We also found that the ERK and JNK signaling pathways were involved in the regulation of IL-8-mediated COX-2 induction in response to HCV infection. Using a promoter-linked reporter assay, we identified that the C/EBP regulatory element within the COX-2 promoter was the dominant factor responsible for the induction of COX-2 by HCV. Silencing C/EBP attenuated HCV-induced COX-2 expression. Our results revealed that HCV-induced inflammation promotes viral replication, providing new insights into the involvement of IL-8-mediated COX-2 induction in HCV replication.

## Introduction

The hepatitis C virus (HCV) is a hepatotropic, non-cytopathic virus that can cause hepatitis, cirrhosis, and hepatocellular carcinoma (HCC) [1]. At present, 170 million people worldwide are chronically infected with HCV [2]. Because HCV can induce chronic hepatic inflammation, it is considered to be one of the major causative factors associated with hepatocellular carcinoma (HCC) development. Many inflammatory cytokines, including TNF-α, TGF-β, interleukin (IL-6), and IL-8, influence cellular signaling and genetic imbalances [3, 4]. HCV core, NS3, and N5A proteins have been reported to result in cytokine imbalance and stimulation of cell growth or suppression of apoptosis for promoting HCC development [5, 6].

Cyclooxygenase (COX) includes the constitutive (COX-1) and inducible (COX-2) isoforms of the COX enzyme for the production of prostanoids (prostaglandins and thromboxanes) [7]. COX-2 is a rate-limiting enzyme, that can be induced by growth factors, tumor promoters, and cytokines and is suggested to be a pathogenic factor involved in inflammation, cellular proliferation, anti-apoptosis activity, and tumorigenesis [8, 9]. Furthermore, increased levels of COX-2 and prostaglandins (PGs) contribute to various biological processes, including acute and chronic inflammation, oxidative stress, bacterial and viral infection, and cancer [10, 11]. In addition, previous reports also demonstrated that the expression of COX-2 was stimulated in response to HCV infection [12, 13]. Notably, significant evidences revealed the enhancement of HCV replication by over-expression of COX-2 [14, 15]. More recently, anti-inflammatory cytokines and an anti-COX-2 signaling pathway were considered as means to inhibit HCV replication and prevent HCV-associated diseases [16]. However, the detailed relationship between HCV and COX-2 has not been elucidated.

IL-8 is a 71-amino acid pro-inflammatory cytokine that belongs to the CXC chemokine family. IL-8 has been demonstrated to influence the chemotaxis of immune cells [17]. Upon receiving inflammatory stimuli, IL-8 can be up-regulated at the transcriptional level in many different cell types, including fibroblasts, monocytes, and hepatocytes, for protecting cells from the aberrant effects of inflammatory stimuli [18]. Several transcription factors have been identified to regulate IL-8, including NF-κB, AP-1, and NF-IL6 [19]. In addition, IL-8 is an important inflammatory mediator in response to viral or bacterial pathogen [20]. Furthermore, IL-8 has the potential to up-regulate some tumor genes, such as cyclooxygenase-2 (COX-2), lipooxygenase-5 (LOX-5), and phospholipase A2 (PLA2), to promote cancer development [21, 22]. The elevated IL-8 expression has been observed in HCV patients [23]. Although the correlations between IL-8 production and sustained virological response (SVR) remain unclear, the IL-8 was considered as the potential biological marker in fibrosis scores and ALT levels [24]. Due to the complicated interaction between inflammation of viral replication, the interaction of HCV replication, IL-8 and COX-2 production need to be further clarified.

In the present study, we demonstrated that HCV infection and HCV NS5A overexpression up-regulate COX-2 expression. We also found that the induction of COX-2 was mediated by HCV-induced IL-8 production. In this context, the transcription factor C/EBP and the ERK/JNK signaling pathway were investigated to determine their roles in the regulation of IL-8-mediated COX-2 expression by HCV.

## Materials and Methods

### Cell cultures and viruses

Ava5 cells, an Huh7 cell line harboring the autonomously replicating HCV subgenomic RNA from NS3 to NS5B region, were provided by Apath (St. Louis, Mo.) [25], naïve Huh7 cells, and a cell culture-produced HCV (HCVcc) infection system [26] were used. Ava5 and naïve Huh7 cells were maintained in complete Dulbecco’s modified Eagle’s medium (DMEM) supplemented with 10% fetal bovine serum (FBS), 1% non-essential amino acids, and 1% antibiotic-antimycotic solution (Life Technologies Co, Ltd., Grand Island, NY). HCVcc was generated by transfection of *in vitro*-transcribed genomic JFH-1 RNA into Huh-7.5 cells according to the method described by Wakita et al [26].

### Plasmid construction

The COX-2 promoter region was amplified from genomic DNA of Huh7 cells as previous described [27]. The different regions of COX-2 promoter were amplified with the corresponding primers; Forward: (-891/+9) 5′-*GGTACC*GGCCATCGCCGCTTCCTTTG-3′, (-362/+9) 5′-*GGTACC*CATCCACGGCGATCAGTCCA-3′, (-193/+9) 5′-*GGTACC*GCAGCTTCCTGGGTTTCCGA-3′, (-96/+9) 5′-*GGTACC*TTGTGGGGGGTACGAAAAGG-3′, and reverse: 5′-*GGTACC*ATGACAATTGGTCGCTAACC-3′, respectively. The each PCR products (-891/+9, -362/+9, -193/+9, and -96/+9) flanked with Kpn I were inserted into the promoterless luciferase vector pGL3-Basic (Promega Co, Madison, WI). The serial COX-2 reporter plasmids with different deleted regions for biding of transcriptional regulator were designed as WT, ΔGRE, ΔGRE/NF-κB, and ΔGRE/ NF-κB /C/EBP. The COX-2 promoter with C/EBP mutant were generated with the primers: forward: 5′-AAAACCCTGCCCCCACCGGCGCGATAGCTTTTTTTAAGGGGAGAGGAG-3′, reverse: 5′-TTTTGGGACGGGGGTGGCCGCGCTATCGAAAAAAATTCCCCTCTCCTC-3′ by QuikChange Site-Directed Mutagenesis Kit according to the manufacturer's protocol (Stratagene, La Jolla, CA). All of the DNA fragments were confirmed by DNA sequencing.

### RNA quantification and western blotting

Total cellular RNA was extracted from cell lysates using a total RNA miniprep purification kit (GMbiolab Co., Ltd, Taiwan) according to the manufacturer’s instructions. cDNA synthesis was performed by M-MLV reverse transcriptase (Promega, WI, USA) according to the manufacturer’s instructions. HCV and inflammatory cytokine RNA levels were detected using quantitative real-time RT-PCR (qRT-PCR) with HCV-specific primers: NS5B, forward: 5′-GGAAACCAAGCTGCCCATCA-3′, reverse: 5′-CCTCCACGGATAGAAGTTTA-3′; TNF-α, forward: 5′-CCTGTGAGGAGGACGAAC-3′, reverse: 5′-AAGTGGTGGTCTTGTTGC-3′; IL-1β, forward: 5′-GGAGAATGACCTGAGCAC-3′, reverse: 5′-GACCAGACATCACCAAGC-3′; iNOS, forward: 5′-CTTTGGTGCTGTATTTCC-3′, reverse: 5′-TGTGACCTCAGATAATGC-3′; and COX-2, forward: 5′-CCGAGGTGTATGTATGAG-3′, reverse: 5′-TGGGTAAGTATGTAGTGC-3′. Each sample was normalized by an endogenous reference gene glyceraldehyde-3-phosphate dehydrogenase (GAPDH) expression. The primers used for GAPDH were as follows: 5′-GTCTTCACCACCATGGAGAA-3′ and reverse: 5′-ATGGCATGGACTGTGGTCAT-3′. Reactions were performed using ABI Step One real-time PCR-system (ABI Warrington, UK). HCV NS5B protein level was determined by Western blotting, which was performed using the standard procedure [27]. The membranes were probed with the following antibodies: anti-NS5B (1:5000; Abcam, Cambridge, MA), anti-COX-2 (1:1000; Cayman, MI, USA), anti-phospho-MPAK (p-ERK1/2, p-p38, and p-JNK), anti-MAPK (ERK1/2, p38, and JNK) (1:1000; Cell Signaling Technology, Inc. Danvers, MA, USA), anti-IL-8, anti-Lamin B, anti-C/EBP (1:1000), and anti-GAPDH (1:10000; GeneTex, CA). Signals were detected using an ECL detection kit (Perkin-Elmer, Branford, CT, USA).

### HCV JFH-1 infection assay

Huh7 cells were seeded in a 24-well plate at a density of 5 × 10^4^ cells/well. After 24-h seeding, the medium was removed and the cells were infected with JFH-1 HCVcc at an MOI of 0.1 for 8 h. At the end of infection, the supernatant was removed and the cells were incubated with fresh medium for an additional 3 days. Upon the completion of inhibitor treatment, HCV RNA and NS5B protein levels were determined using qRT-PCR and western blotting, respectively.

### Measurement of IL-8 and production

Huh7 cells were seeded in a 24-well plate at a density of 5 × 10^4^ cells/well. The cells were then transfected with the indicated concentrations of expression vector for 8 h. The transfected cells were incubated with or without JFH-1 HCVcc at an MOI of 0.1 for an additional 8 h. The supernatant was collected at the indicated time points and used to determine IL-8 levels using the IL-8 ELISA assay kit according to the manufacturer’s instructions (Millipore Corporation, Billerica, MA, USA).

### Measurement of PGE_2_ and production

Huh7 cells were seeded in 48-well plates at a density of 5 × 10^3^, and then transfected with the indicated expression vector. After 3 days incubation, the cell were collected and the cell membranes were lysed to release intracellular PGE_2_. The amounts of PGE_2_ production were analyzed with the PGE_2_ enzyme-linked immunosorbent assay system (Biotrak, Amersham Bioscience) according to the Manufacturer’s protocol.

### Transfection and Luciferase activity assay

To evaluate the regulation of COX-2 and IL-8 expressions by NS5A, Huh7 and Ava5 cells were transfected with different concentrations of pCMV-NS5A-Myc or control vector pcDNA4/myc (mock control) by T-Pro reagent (Ji-Feng Biotechnology Co., Ltd. Taiwan) according to the manufacturer’s instructions. After 3 days of incubation, the lystes were harvested and the expressions were quantified by western blot and qRT-PCR. The “Mock” control represented as the vehicle control transfected with the empty vector, pcDNA4-myc. To evaluate the regulation of COX-2 expression by HCV, Huh7 and Ava5 cells were transfected with 1 μg of pCOX-2-Luc, pCOX-2(ΔGRE)-Luc, pCOX-2(ΔGRE/NFkB)-Luc, pCOX-2(ΔGRE/NFkB/C/EBP)-Luc, or pCOX-2(ΔGRE/NFkB-C/EBP^mut^)-Luc reporter plasmid. To evaluate the role of IL-8 on COX-2 expression, Huh7 cells were transfected with increasing concentrations of the IL-8 shRNA expression vector (pIL-8-shRNA; 0.25–2 μg) in the presence of JFH-1 infection. All transfection reactions contained 0.1 μg of a secreted alkaline phosphatase (SEAP) expression vector (pSEAP) for normalization of transfection efficiency in each reaction. After 3 days of incubation, the supernatants were harvested for the SEAP activity assay with the Phospha-Light assay kit (Tropix, Foster City, CA, USA) and the cell lysates were subjected to the luciferase activity assay with the Bright-Glo luciferase assay system (Promega, Madison, WI, USA) in accordance with the manufacturer’s instructions for measuring the relative COX-2 promoter activity.

### Preparation of nuclear extract

Huh7 cells were seeded in a 6-cm dish at a density of 4 × 10^5^ cells. At indicated times of incubation, the nuclear and cytoplasm extracts were separated and isolated using hypotonic and high-salt buffer extraction with protease inhibitors and phosphotase inhibitors, as described previously [28]. The cells were lysed using ice-cold hypotonic buffer (10 mM HEPES, 1.5 mM MgCl_2_, 10 mM KCl, 0.5 mM DTT, 10% Nonidet P-40, pH 7.9) with centrifugation at 7000 × *g* for 15 min. The nuclear pellets were incubated with a high-salt nuclear extraction buffer (20 mM HEPES, 1.5 mM MgCl_2_, 0.2 mM EDTA, 0.6 M KCl, 0.2 mM DTT, 0.5 mM DTT, pH 7.9) and vortexed within 40 min. During the final steps of separation, the nuclear extracts were collected by centrifugation at 20000 × *g* for 15 min. All the collected lysates were stored at −80°C until use.

### Statistical Analysis

The results were reported as the means ± SD for at least 3 independent experiments. Statistical significance of multiple experimental group was analyzed using ANOVA by GraphPad Prism Software (Graphpad Software, Inc, San Diego, CA). *p < 0.05 or **p < 0.01 indicated statistical significance.

## Results

### 1. HCV proteins induce COX-2 expression

**Fig 1:**
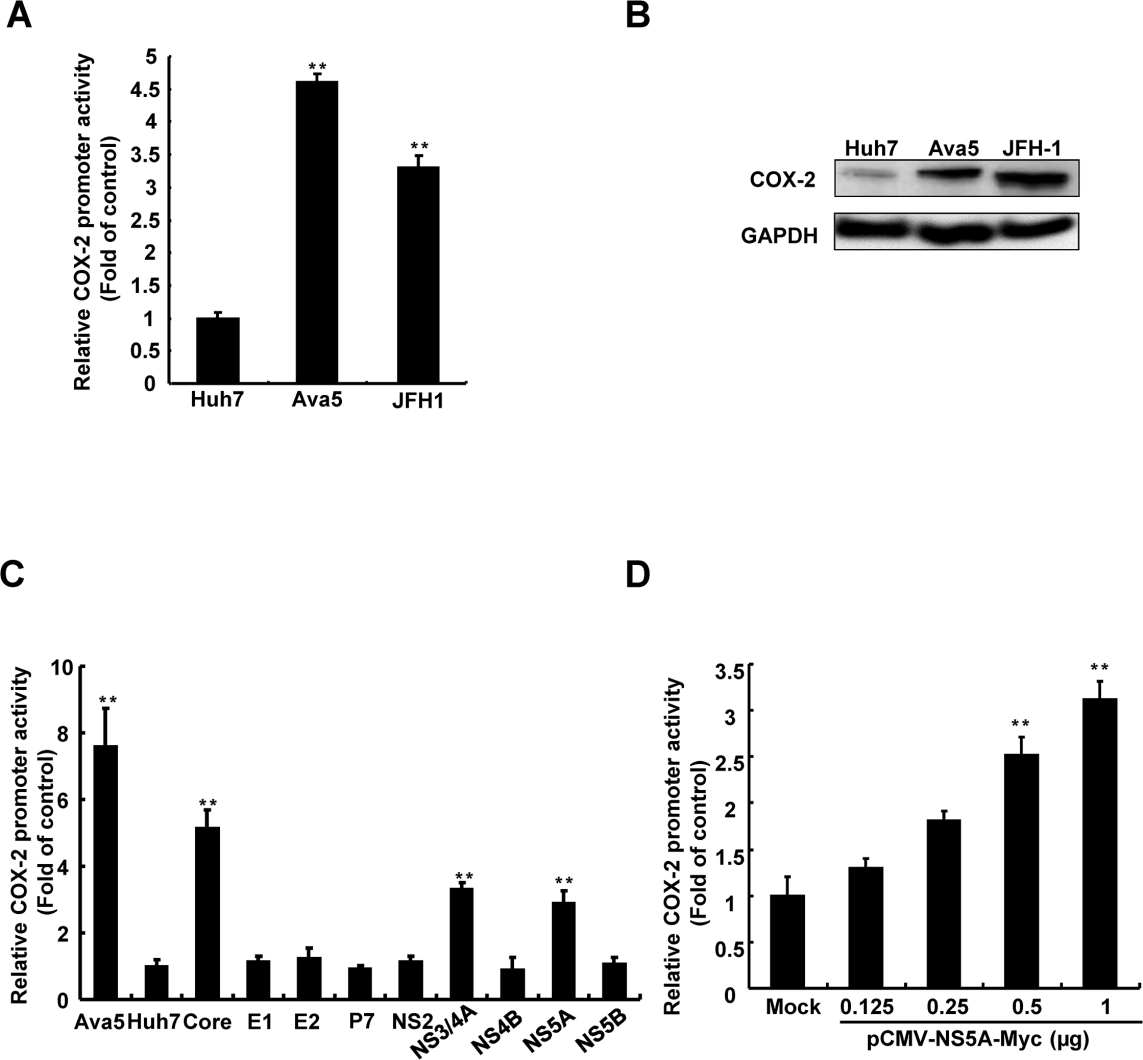
Determination of the effect of HCV on COX-2 expression. (A, B) Regulation of COX-2 expression in hepatoma cells is mediated by HCV. pCOX-2-Luc was transfected into Huh7, Ava5, and JFH-1-infected Huh7 cells to measure the effect of COX-2 on HCV protein expression. The cell lysates of Huh7, Ava5 (harboring HCV subgenomic replicon), and JFH-1-infected Huh7 cells were subjected to western blotting. (C) Identification of the viral proteins of HCV that mediate COX-2 regulation. Huh7 cells were co-transfected with pCOX-2-Luc and different viral protein expression vectors (0.5 μg). (D) HCV NS5A induces COX-2 transcriptional activity. Huh7 cells were co-transfected with pCOX-2-Luc and different amounts of pCMV-NS5A-Myc (0–2 μg). After incubation for 3 days, the cell lysates were subjected to the luciferase activity assay to measure the induction of the COX-2 promoter. The activity of Ava5 cells was the positive control. “Mock” indicates the co-transfection of empty vector, pcDNA4/myc, and pCOX-2-Luc. Data shown are mean ± SE; n = 3. *p < 0.05. **p < 0.01.

To investigate COX-2 activation regulated by HCV infection, we first examined COX-2 expression in HCV-infected Huh7 hepatoma cells and Ava5 cells, Huh7 cell line carrying consistent subgenomic HCV protein expression, using a COX-2 promoter-based reporter assay. Parental Huh7, Ava5, or JFH-1-infected Huh7 cells were transfected with the pCOX-2-FLuc reporter plasmid. The resulting cell lysates were analyzed using luciferase assay and western blotting. As shown in Fig 1A and 1B, significant induction of COX-2 promoter activity and protein expression were observed following HCV infection and HCV subgenomic protein expression. To further determine which HCV proteins are involved in COX-2 activation, we co-transfected pCOX-2-FLuc with each HCV protein expression vector into Huh7 cells and subsequently measured firefly luciferase activity. As shown in Fig 1C, we observed that compared with parental Huh7 cells, transfected cells producing HCV core, NS3, and NS5A induced significantly more COX-2 promoter activity. The COX-2 promoter-based reporter assay further demonstrated that NS5A induced COX-2 promoter activity in a concentration-dependent manner (Fig 1D). Previous studies have demonstrated that HCV core and NS3 induced COX-2 expression [29, 30]; however, there have been few studies on COX-2 expression elevated by NS5A. Therefore, it is worthwhile to evaluate the effect of NS5A on COX-2 regulation.

### 2. HCV infection and NS5A protein up-regulate the pro-inflammatory cytokine IL-8

**Fig 2:**
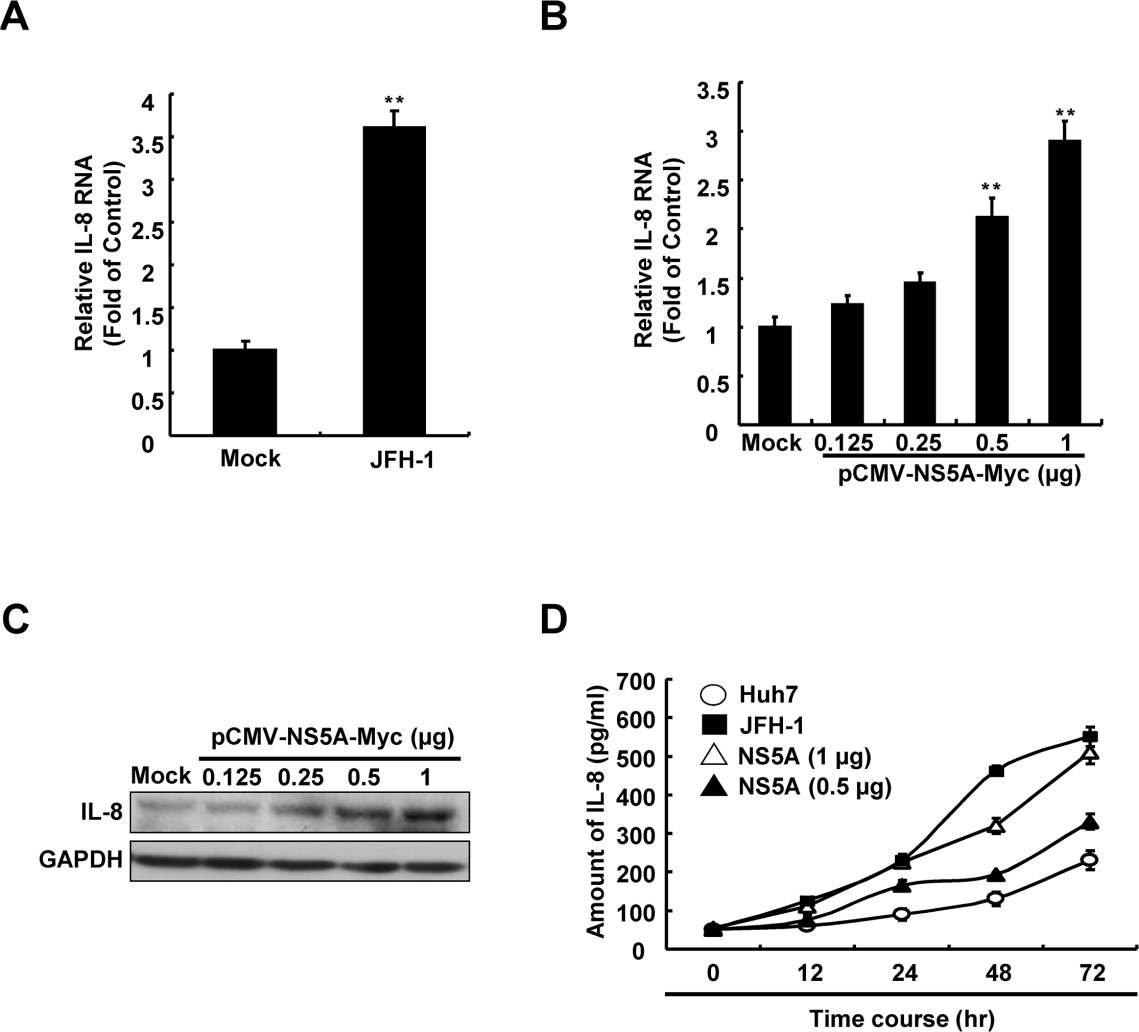
Elucidation of the regulation of IL-8 expression in hepatoma cells with HCV infection and HCV NS5A. (A) Induction of IL-8 transcription by HCV infection and HCV NS5A expression. Huh7 cells were infected with JFH-1 and transfected with pCMV-NS5A-Myc. After 3 days, the total cellular RNA was extracted to quantify the relative RNA levels of IL-8 normalized to *gapdh* expression using qRT-PCR. (B) Induction of IL-8 production by HCV NS5A expression. Huh7 cells were transfected with pCMV-NS5A-Myc and the relative RNA and protein levels of IL-8 were analyzed by using qRT-PCR and western blot. (C) After 3 days, the cell supernatant was analyzed to quantify the amount of IL-8 using an ELISA kit, as described in the Materials and Methods. Mock control indicates Huh7 cells transfected with the vehicle vector. Data shown are mean ± SE; n = 3. *p < 0.05. **p < 0.01.

To identify the effects of HCV infection on IL-8 expression, we first monitored IL-8 expression in JFH-1-infected Huh7 cells using qRT-PCR. As shown in Fig 2A, HCV infection significantly increased IL-8 RNA levels. Further, to identify whether IL-8 is up-regulated by HCV NS5A, we transfected Huh7 cells with an HCV NS5A expression vector, pCMV-NS5A-Myc (0.125–1 μg). After 3 days, total cellular RNA and cell lysates were collected and the relative IL-8 expression levels were analyzed using qRT-PCR and western blotting, respectively. As shown in Fig 2B and 2C, compared with parental Huh7 cells, overexpression of HCV NS5A in JFH-1-infected Huh7 cells resulted in a concentration-dependent increase in both RNA and protein levels of IL-8. Subsequently, the influence of HCV infection or NS5A overexpression on the production of IL-8 was evaluates by measuring the levels of IL-8 in the supernatant using an ELISA assay. As shown in Fig 2D, the levels of IL-8 increased in a time-dependent manner in HCV-infected or NS5A-transfected Huh7 cells compared with those of parental Huh7 cells, indicating that HCV NS5A correlated with the induction of IL-8.

### 3. HCV up-regulates COX-2 expression via IL-8

**Fig 3:**
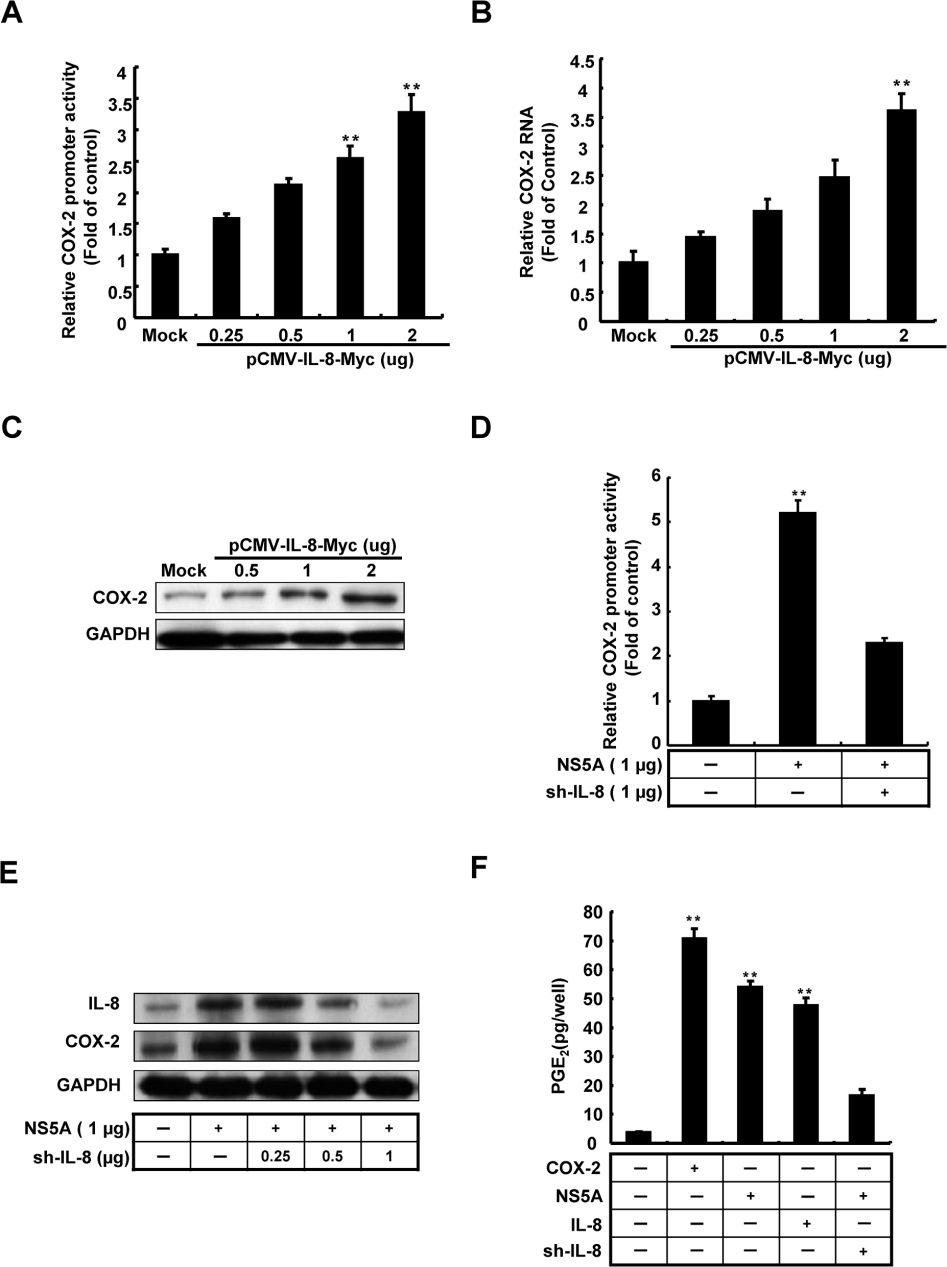
Analysis of the role of IL-8 in the regulation of COX-2 expression mediated by HCV. Induction of COX-2 (A) promoter activity, (B) RNA levels, (C) protein expression in a dose-dependent manner. The Huh7 cells were co-transfected with pCOX-2-Luc and different amounts of pCMV-IL-8-Myc (0–2 μg). After incubation for 3 days, the cell lysates were subjected to luciferase activity assay to measure the induction of COX-2 promoter by Steady-Glo luciferase assay system. The Huh7 cells were transfected with different amounts of pCMV-IL-8-Myc (0–2 μg). After 3 days incubation, the total RNA and cell lysates were extracted and analyzed by qRT-PCR and western blot, respectively. GAPDH shows the loading control. (C, D) Determination of the effect of IL-8 shRNA on COX-2 expression by NS5A. Huh7 cells were co-transfected with pCOX-2-Luc, pCMV-NS5A-Myc (1 μg), and different amounts of IL-8 shRNA (0–1 μg). After 3 days of incubation, the total cell lysates were extracted and analyzed using the luciferase assay and western blotting. (F) Determination of the effect of HCV and IL-8 on PGE_2_ expression. The Huh7 cell were transfection with pCMV-NS5A-Myc combined with or without sh-IL-8 or treated with recombinant IL-8. The Huh7 cells transfected with pCMV-COX-2-Myc served as a positive control of PGE_2_ production. After 3 days, the intercellular PGE_2_ levels were assayed with the Biotrak PGE_2_ enzyme immunoassay system. The relative IL-8 and COX-2 RNA levels were normalized by *gapdh* expression. GAPDH was used as an equal loading control. Data shown are mean ± SE; n = 3. *p < 0.05. **p < 0.01.

HCV infection and viral protein expression are accompanied by acute inflammation and cytokine production, leading to hepatocarcinogenesis. Therefore, we focused on the relation between induction of COX-2 expression and IL-8 production. Because of there is not much information on the relevance of their co-expression from HCV clinical findings, we queried that whether IL-8 activated COX-2 expression. To identify whether the transactivities of COX-2 were induced with IL-8 over-expression, Huh7 cells were co-transfected with IL-8 expression vectors, pCMV-IL-8-Myc, and pCOX-2-Luc. As shown in Fig 3A, IL-8 significantly induced COX-2 promoter activity in a dose-dependent manner. To verify the regulation of COX-2 expression by IL-8, we transfected pCMV-IL-8-Myc in Huh7 cells and the total cellular RNA and cell lysates were collected after 3 days. The COX-2 expressions were analyzed by qRT-PCR and western blot, respectively. As shown in Fig 3B and 3C, we found that increasing concentrations of IL-8 significantly induced COX-2 RNA level and protein expression, which corroborated the results of the promoter activity assay. To investigate whether there is a correlation between HCV, IL-8, and COX-2, we performed a COX-2 promoter-based reporter assay to examine the effects of knock-down of IL-8 on NS5A-induced COX-2 expression. Huh7 cells were co-transfected with the reporter vector pCOX-2-Luc and NS5A expression vector pCMV-NS5A-Myc in the presence or absence of the effective IL-8 shRNA expression vector. As shown in Fig 3D, NS5A-induced COX-2 promoter activity was significantly reduced when IL-8 expression was knocked-down. Consistent with these data, the results of western blotting revealed that IL-8 knock-down attenuated NS5A-induced COX-2 protein expression in a concentration-dependent manner (Fig 3E). To identify whether IL-8 involved in PGE_2_ production, the Huh7 cells were treated with recombinant IL-8 or co-transfected with pCMV-NS5A-Myc with or without IL-8 shRNA, respectively. The over-expressed COX-2 served as positive control for PGE_2_ production. As shown in Fig 3F, we observed IL-8 significant induced PGE_2_ production. These results suggested that IL-8 participates in the regulation of COX-2. Therefore, we suggest that IL-8 is involved in HCV-induced COX-2 expression.

### 4. The ERK/JNK pathway is involved in HCV-induced COX-2 expression

**Fig 4:**
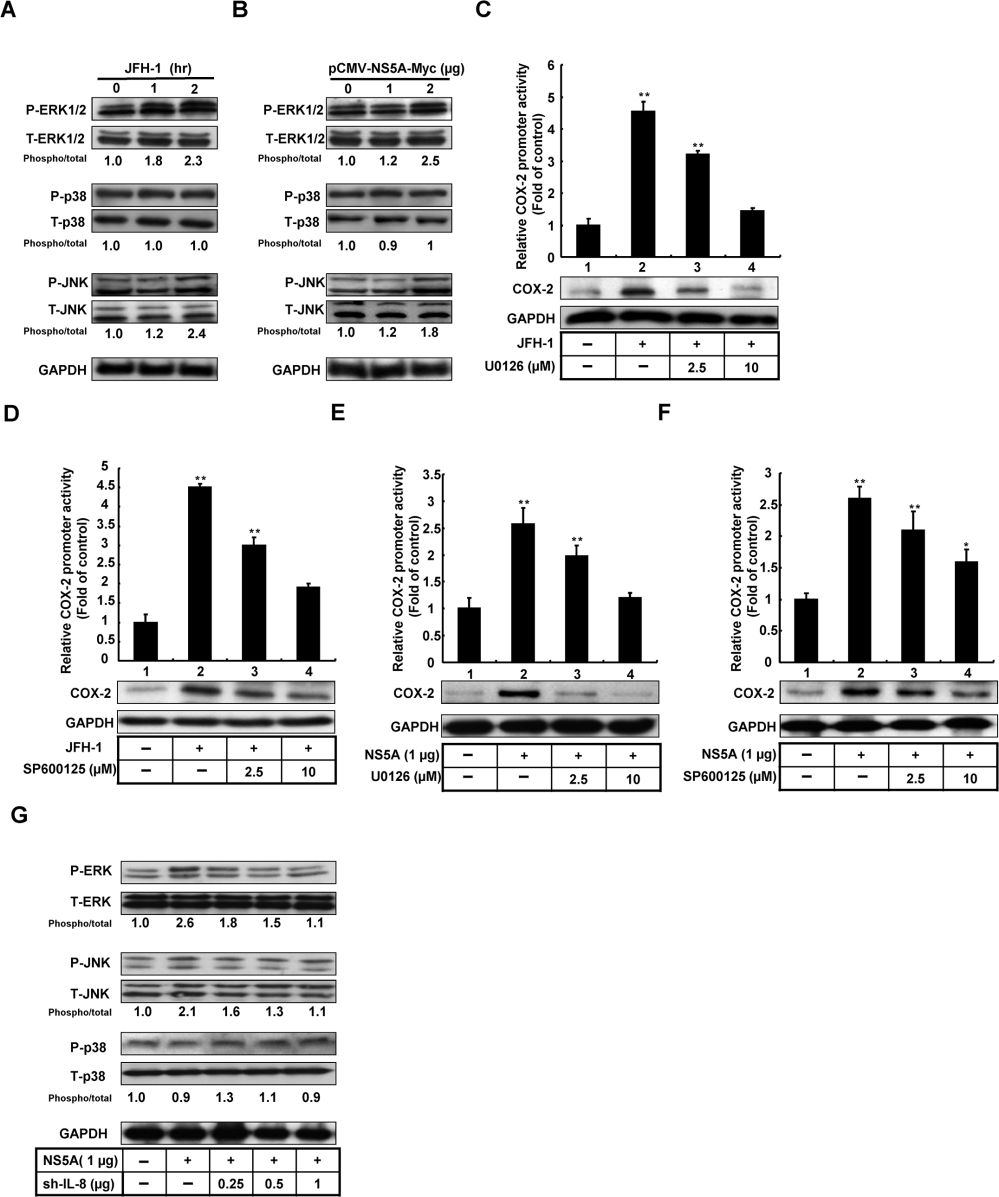
Analysis of the effects of ERK and JNK signaling pathways on COX-2 expression mediated by HCV. (A, B) Huh7 cells were infected with HCV JFH-1 or transfected with pCMV-NS5A-Myc. Following infection or transfection, the cells were grown in complete medium. The cell lysates were collected and subjected to western blotting with specific antibodies for MAPK (ERK, p38, and JNK) and phospho-MAPK. (C-F) To identify the COX-2 activation through ERK and JNK activation upon HCV NS5A expression or HCV infection, Huh7 cells were co-transfected with pCOX-2-Luc with pCMV-NS5A-Myc or infected with HCV JFH-1. Following transfection or infection, the cells were grown in complete medium with or without the specific ERK inhibitor U0126 and JNK inhibitor SP600125. The cell lysates were collected and subjected to the luciferase activity assay to measure COX-2 promoter activity and western blotting with antibodies specific for COX-2 and GAPDH. (G) Huh7 cells were co-transfected with pCMV-NS5A-Myc (1 μg), and different amounts of IL-8 shRNA (0–1 μg). After 3 days, the cell lysates were collected and subjected to western blotting with antibodies specific for MAPK (ERK, p38, and JNK) and phospho-MAPK. GAPDH was used an equal loading. Data shown are mean ± SE; n = 3. *p < 0.05. **p < 0.01.

Previous studies have shown that activation of the MAPK pathway plays an important role in HCV replication and HCV-related inflammation response [31]. To identify the role of the MAPK pathway in COX-2 regulation upon HCV infection and HCV NS5A expression, we analyzed the relative phosphorylation levels of ERK, JNK, and p38 in HCV-infected and NS5A-transfected Huh7 cells by western blotting. A time-dependent increase in the phosphorylation levels of ERK and JNK was observed in HCV-infected Huh7 cells (Fig 4A). Similarly, a concentration-dependent increase in the phosphorylation levels of ERK and JNK was observed in NS5A-transfected Huh7 cells (Fig 4B). Furthermore, we also observed that the phosphorylation levels of ERK and JNK was increased at 2 hrs in response to 100 pg/ml of IL-8, an equal concentration of IL-8 stimulated by HCV JFH-1 infection (S1A Fig). However, there is no significant difference of IL-8 production with JFH-1 infection in the presence of specific inhibitors of ERK and JNK treatment (S1B Fig). Therefore, IL-8 production play an important role in the regulation of HCV-mediated COX-2 expression. To identify the role of the ERK and JNK pathways in COX-2 regulation following HCV infection, HCV-infected Huh7 cells were transfected with the reporter plasmid pCOX-2-Luc and then treated with specific inhibitors of ERK and JNK. As shown in Fig 4C and 4D, JFH-1-induced COX-2 promoter activity and COX-2 expression were significantly reduced following ERK and JNK inhibitor treatment (columns 3 and 4) compared with untreated HCV-infected Huh7 cells (column 2) and parental Huh7 cells (column 1). Similarly, ERK or JNK inhibitor treatment significantly attenuated HCV NS5A-enhanced COX-2 promoter activity and COX-2 expression (Fig 4E and 4F). To further clarify the effect of IL-8 on the NS5A-mediated MAPK signaling pathway, we compared the levels of active phospho-MAPK molecules in pCMV-NS5A-Myc and IL-8 shRNA co-transfected Huh7 cells using western blotting. As shown in Fig 4G, IL-8 knock-down resulted in a reduction of NS5A-enhanced ERK and JNK phosphorylation levels in a concentration-dependent manner. Based on these results, we concluded that IL-8-mediated COX-2 expression was induced by an increase in the phosphorylation levels of ERK and JNK by HCV.

### 5. IL-8-mediated COX-2 expression with HCV infection was induced by C/EBP transcription factor

**Fig 5:**
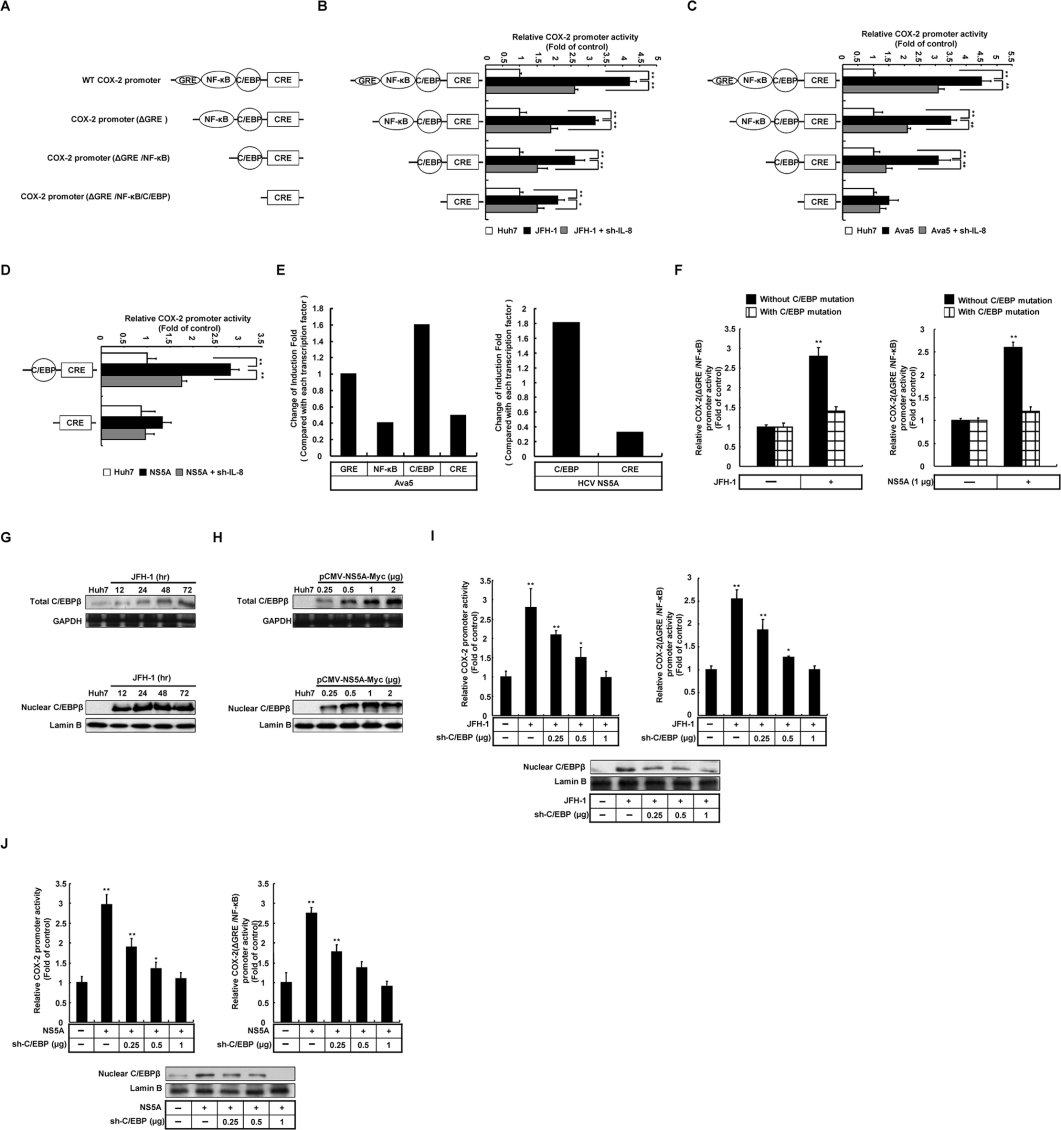
Determination of the effects of C/EBP on COX-2 expression regulated by HCV. (A) Diagrams of COX-2 promoter with series deletion containing binding element of transcription factor, which includes the wild-type COX-2 promoter (WT, −891/+9) and series deletion (ΔGRE, ΔGRE/NF-κB, ΔGRE/ NF-κB /C/EBP). (B, C) Huh7, JFH-1 infected Huh7, and Ava5 cells were co-transfected with each of the COX-2 reporter clones for analyzing the induction of COX-2 transcription factors. (D) The COX-2 reporter vector, pCOX-2(ΔGRE/NF-κB)-Luc or pCOX-2(ΔGRE/NF-κB/C/EBP)-Luc (1 μg) was transfected into Huh7 cells with or without pCMV-NS5A-Myc. After 3 days of incubation, the cell lysates were subjected to the luciferase assay to measure the induction of COX-2 promoter activity. (E) The each transcription factor influenced induction fold change of COX-2 promoter in Ava5 cells or HCV NS5A-expression Huh7 cells. (F) Huh7 cells were transfected with pCOX-2(ΔGRE/NF-κB-C/EBP^mut^)-Luc with NS5A overexpressing and JFH-1-infected cells. The promoter activity was assayed by the luciferase system. (G, H) Huh7 cells were transfected with pCMV-NS5A-Myc or infected with HCV JFH-1 and the total expression (upper panel) and nucleus translocation level (lower panel) of C/EBP was analyzed by western blotting. (I, J) Huh7 cells were co-transfected with pCOX-2-Luc with pCMV-NS5A-Myc or infected with HCV JFH-1 combined with C/EBP knock-down. The cell lysates were collected and subjected to western blotting and luciferase assay analysis, respectively. Lamin B1 and GAPDH were used as an equal loading controls. Data shown are mean ± SE; n = 3. *p < 0.05. **p < 0.01.

To identify the transcription factors involved in IL-8-mediated COX-2 expression in HCV infection, we constructed several COX-2 reporter plasmids containing serial deletions of the COX-2 promoter fragment, including WT, ΔGRE, ΔGRE/NF-κB, and ΔGRE/NF-κB /C/EBP, linked to the firefly luciferase reporter gene for the reporter assay (Fig 5A). In addition, to investigate COX-2 promoter activity regulated by HCV-induced IL-8 production, we co-transfected each reporter plasmid with IL-8 shRNA in Huh7 cells with and without JFH-1 infection (MOI = 0.1) for 3 days. Further, in the HCV replicon system, a similar experimental procedure was also performed in Ava5 cells. The relative COX-2 promoter activities were analyzed by the luciferase assay. As shown in Fig 5B and 5C, as expected, the WT COX-2 promoter activities were significantly elevated in HCV-infected and Ava5 replicon cells compared with parental Huh7 cells. In addition, a significant induction of the promoter activity of the construct with GRE, NF-κB, and C/EBP binding sites, but no significant difference was observed with only the CRE binding site in HCV-infected Huh7 and Ava5 replicon cells. Collectively, these results reveal that C/EBP recognition site is involved in the activation of COX-2. Comparing the reporter assay data, we also observed that the HCV-induced COX-2 promoter activity containing the C/EBP binding site showed a significant reduction with IL-8 knock-down. Therefore, we suggested that C/EBP may play a dominant role in IL-8-mediated COX-2 induction with HCV protein expression. Based on previous results, we suggested that HCV NS5A is responsible for COX-2 induction in HCV infection and replication. To determine whether HCV NS5A induces COX-2 promoter activity through C/EBP activation, Huh7 cells were co-transfected with pCOX-2(ΔGRE/NF-κB)-Luc, pCOX-2(ΔGRE/NF-κB/C/EBP)-Luc, and pCMV-NS5A-Myc. Simultaneously, the promoter activities were also confirmed with IL-8 knock-down. As shown in Fig 5D, the COX-2 promoter activity containing the C/EBP binding site was significantly elevated by NS5A over-expression and reduced with IL-8 knock-down. In contrast, the CRE binding activity with NS5A overexpression showed no significant difference compared with parental Huh7 cells. Despite some noise interfered the COX-2 promoter activity by structural proteins under JFH-1 infection (S2 Fig), we found the most significant activation of COX-2 promoter contain C/EBP binding site with NS5A-expressing Huh7 cells and in replicon cells by comparison of relative change of induction fold of reporter activity (Fig 5E). To verify whether the HCV-induced COX-2 promoter activity was dependent on C/EBP activation, we constructed a reporter plasmid carrying a point mutation in C/EBP consensus sequence and transfected this reporter plasmid into Huh7 cells in the presence of NS5A expression or HCV infection. After 3 days, we found that there was no significant induction of COX-2 promoter activity with the mutation of the C/EBP binding site by HCV (Fig 5F). To confirm the results of the reporter assay, we examined whether C/EBP expression was regulated by HCV infection and HCV NS5A expression. Huh7 cells were infected with JFH-1 or transfected with the HCV NS5A expression vector pCMV-NS5A-Myc (0.125–1 μg) for 3 days. C/EBP expression and efficiency of nucleus translocation were analyzed by western blotting. As shown in Fig 5G and 5H, we found significant C/EBP induction and translocation of C/EBP from the cytosol to the nucleus upon HCV infection and HCV NS5A overexpression. Further, the HCV-induced COX-2 promoter activity was significantly reduced with C/EBP knock-down by specific shRNA (Fig 5I and 5J). Furthermore, we also found that IL-8 induced-PGE_2_ production was attenuated with C/EBP knock-down (S3 Fig). Taken together, these results clearly demonstrate that HCV-induced COX-2 expression depends on IL-8-mediated C/EBP activation.

### 6. IL-8 knock-down influences the inflammatory response induced by HCV

**Fig 6:**
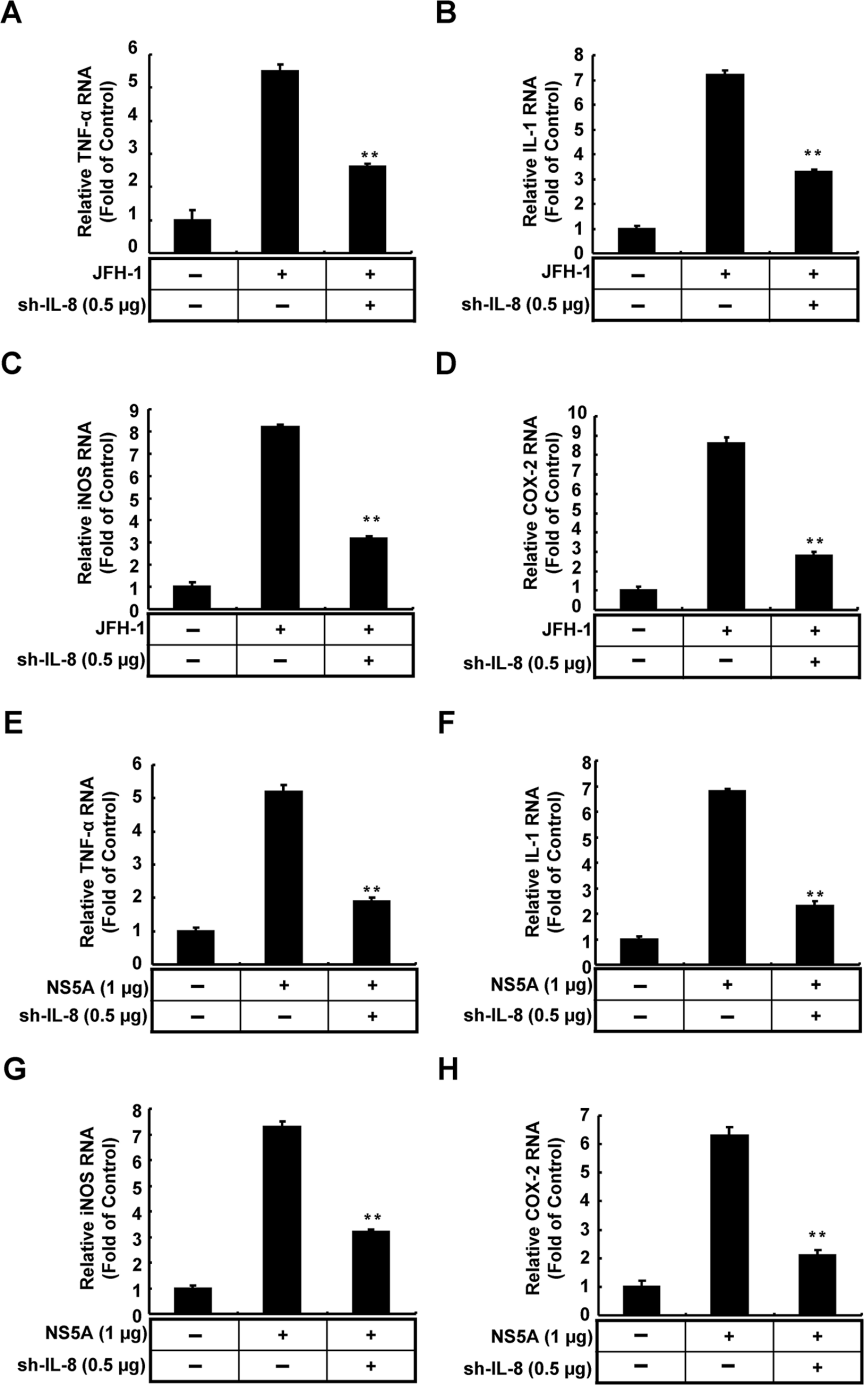
Determination of the effects of IL-8 knockdown on inflammatory cytokine production mediated by HCV NS5A expression and HCV infection. (A-D) Huh7 cells were transfected with IL-8 shRNA expression vector (0.5 μg) and then infected with HCV JFH-1 for 6 h. After 3 days of incubation, the total cellular RNA was extracted and analyzed using qRT-PCR. (E-H) Huh7 cells were co-transfected with pCMV-NS5A-Myc (1 μg) and IL-8 shRNA expression vector (0.5 μg). After 3 days of incubation, the total cellular RNA was extracted and analyzed using qRT-PCR. The relative TNF-α, iNOS, IL-1, and COX-2 RNA levels were normalized by *gapdh* expression. Data shown are mean ± SE; n = 3. *p < 0.05. **p < 0.01.

IL-8 has been identified as a key component in the different stages of cancer development, including inflammation, tumor proliferation, metastasis, and angiogenesis [32]. To examine whether IL-8 participates in inflammatory responses upon viral infection or viral protein expression, we measured the effects of IL-8 knock-down on the mRNA levels of several inflammatory mediators, including TNF-α, iNOS, COX-2, and IL-1, in JFH-1-infected or NS5A-expressing cells using qRT-PCR. Parental Huh-7 cells and IL-8 shRNA-transfected cells were infected with JFH-1 (MOI = 0.1) or co-transfected with NS5A and then cultured in complete medium for an additional 3 days. The resulting total cellular RNAs were collected and analyzed by qRT-PCR using specific paired primers of individual cytokines. As shown in Fig 6, HCV infection (A–D) and NS5A overexpression (E–H) both elevated the mRNA levels of the inflammatory mediators compared with the levels in parental cells, and IL-8 knock-down resulted in a significant reduction of expressions of these inflammatory mediators. The results of qRT-PCR revealed that IL-8 has a potential role in HCV-associated liver inflammation or HCC development.

### 7. IL-8-mediated COX-2 induction assists in HCV replication

**Fig 7:**
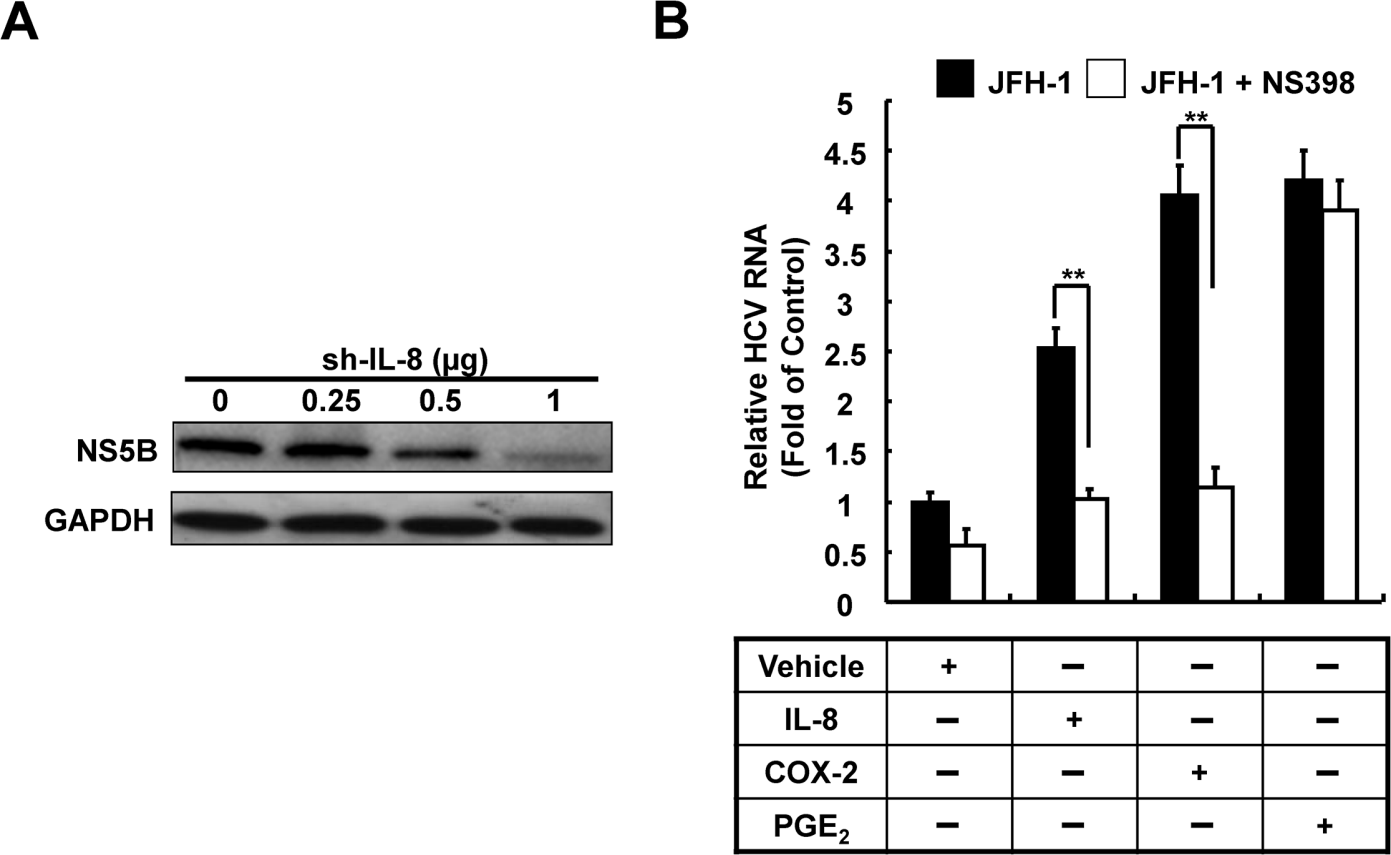
Identification of the roles of IL-8 and COX-2 in HCV replication. (A) Ava5 cells were transfected with IL8-shRNA in a concentration-dependent manner (0–2 μg). After 3 days of incubation, the total cell lysates were extracted and HCV protein synthesis was analyzed by western blotting. GAPDH was used as the equal loading control. (B) Huh7 cells were transfected and treated with pCMV-NS5A-Myc, pCMV-COX-2-Myc, recombinant IL-8 protein, and PGE_2_. The transfected and treated cells were incubated with or without NS398 for 3 days. The relative HCV RNA levels were analyzed using qRT-PCR. The HCV RNA level of JFH-1-infected Huh7 cells was defined as 1. Data shown are mean ± SE; n = 3. *p < 0.05. **p < 0.01.

Previous studies have shown that COX-2 plays an important role in HCV replication; accordingly, we proposed that HCV-induced IL-8 production exerts a positive feedback effect during HCV replication. First, we sought to identify the role of IL-8 in HCV replication. Ava5 cells were transfected with different concentrations of IL-8 shRNA for 3 days. The cell lysates were collected and HCV protein expression levels were analyzed by western blotting. As shown in Fig 7A, we found that the synthesis HCV proteins was significantly reduced with increasing concentrations of IL-8 shRNA. To further determine whether HCV can auto-regulate its replication through IL-8-mediated COX-2 induction, Huh7 cells were infected with a low MOI of JFH-1 (MOI = 0.05) and observed for enhancement of replication. Subsequently, the infected cells were treated with recombinant IL-8 protein or PGE_2_ combined with or without the COX-2 inhibitor (NS398), respectively. In addition, Huh7 cells were transfected with pCMV-COX-2-Myc. Subsequently, the transfected cells were infected with JFH-1 (MOI = 0.05) and treated with or without the COX-2 inhibitor (NS398) that served as the positive control for COX-2 induction. After 3 days, the total cellular RNA was collected and relative HCV RNA levels were analyzed using qRT-PCR. As shown in Fig 7B, we found that COX-2 stimulation and COX-2 overexpression significantly increased HCV replication, whereas treatment with increasing concentrations of COX-2 inhibitors reduced the viral replication potential. In addition, JFH-1-infected cells treated with PGE_2_ showed a significant induction of HCV replication with NS398 treatment. Taken together, we concluded that HCV-induced IL-8 production exerts a positive feedback effect on HCV replication through IL-8-mediated COX-2 expression.

## Discussion

Many studies have reported the activation of COX-2 expression during viral infection, In addition, the clinical evidence show significant correlation between COX-2 expression and HCC[33]. In the present study, we demonstrated that HCV infection induced COX-2 expression through MAPK pathway (ERK and JNK) activation with viral-induced IL-8. Following a viral stimulus or cell stresses, several transcription factors have been found responsible for activating COX-2 induction. In many cell types, including neurons, skin cells, or macrophages, COX-2 expression is significantly induced under stress, with the up-regulation of NF-κB, AP-1, CREB, and C/EBP[34]. In this study, we also showed that C/EBP is a dominant transcription factor that induces COX-2 following HCV infection and NS5A overexpression through IL-8 induction in liver cells.

**Fig 8:**
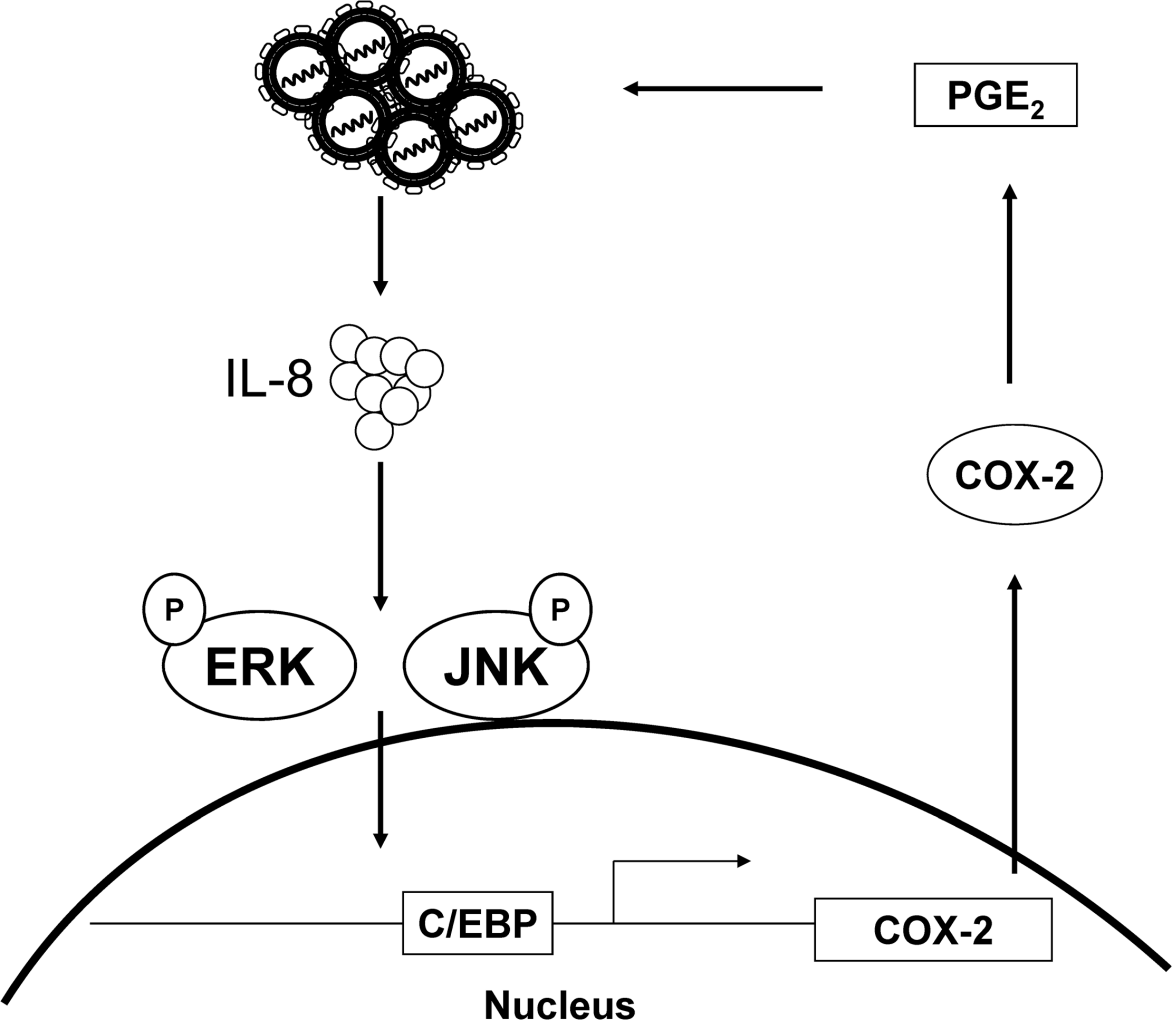
A proposed model of COX-2 up-regulation by HCV infection.

The inflammatory response is an early stage of viral infection; the late stage of infection and prolonged inflammation can result in liver damage [35]. In addition, HCV infection and HCV proteins influence the immune system and cytokine production. Previous studies have supported a correlation between IL-8 expression levels and angiogenesis in HCC [36, 37]. With the clinical data, the serum levels of IL-8 from HCV patients were proposed as a biomarker for the prediction of the tumor size or risk of developing HCC [38]. With regard to the association between HCV-induced inflammation and carcinogenesis, we found a reduction in the expression levels of different cytokine with IL-8 knock-down (Fig 6). Chronic HCV infection, as well as overexpression of certain proteins, increases the levels of IL-8, which is in turn is correlated with COX-2 induction (Figs 2 and 3). Hence, we propose a model of HCV up-regulating COX-2 expression via IL-8-mediated activation of the ERK/JNK MAPK pathway (Fig 8). Further, we also found that IL-8 production influences the expression of immune cytokines, including IL-1, TNF-α, iNOS, and COX-2, which shows the importance of the inflammatory environment of liver carcinogenesis. Therefore, we suggest that HCV-induced IL-8 production plays an essential role in HCC development and HCV replication.

Although many studies have reported that COX-2 is regulated by several transcription factors, the intracellular signaling pathway in Huh7 with HCV infection remains unclear. In a promoter-based assay, we discovered that the commonly induced transcription factors with HCV infection and NS5A overexpression are NF-κB and C/EBP. Furthermore, we found that the significant induction of the phosphorylation level of C/EBP and the C/EBP-dependent activation of COX-2 promoter by HCV (Fig 5). In general, C/EBP activation is considered an important factors promoting liver fibrosis [39]. In this regard, it is noteworthy that our results are consistent with the findings of overexpression of C/EBP and COX-2 in the liver biopsy data from patients with HCC [40] and *in vivo* study [41]. In addition, both overexpression of NS5A and activation of C/EBP affect the insulin pathway, lipid modulation, and liver steatosis [42]. The detailed correlation between HCV and C/EBP in clinical findings remains unclear. We suggest that NS5A-induced C/EBP activation has a potential role in the insulin resistance of patients with HCV and that it may be involved in viral-induced IL-8 production.

Viral infection has been considered to be responsible for inducing oxidative stress, which is accompanied by the generation of reactive oxygen species [17] and modulation of biological molecules that affect gene expression and cell metabolism [43]. Chronic infection may lead to constitutive induction of COX-2 expression, which promotes the synthesis of PGE_2_ to enhance STAT3 phosphorylation and the regulation of tumor growth [44]. The results of several studies have revealed that PGE_2_ activation promotes HCC cell proliferation and invasion [41, 45].Several studies have shown that the MAPK signaling pathway participates in HCV replication and related carcinogenesis; these findings emphasize the role of kinases in COX-2 regulation. We observed the phosphorylation of ERK and JNK at much earlier time points than that of p38 in JFH-1-infected and NS5A-overexpressing cells (Fig 4). However, the phosphorylation of ERK and JNK by HCV was blocked by pre-treatment with specific inhibitors. COX-2 activity and protein synthesis were reduced with increasing concentrations of inhibitors. Furthermore, in conjunction with IL-8 knock-down, the phosphorylation of ERK and JNK in HCV-infected and NS5A-expressing cells was also reduced. Our results demonstrated that the phosphorylation of ERK and JNK is involved in COX-2 regulation by IL-8 and IL-8 knock-down abolishes the kinase phosphorylation level, COX-2 promoter activity, and protein synthesis.

Several studies have shown that host factors participate in HCV replication, and COX-2 expression regulates HCV replication in Huh7 cells [14, 15]. In our previous studies, we also identified COX-2 as a potential target for anti-HCV drug development [27, 46]. Previously, Waris and Siddiqui's studies revealed the elevated COX-2 and PGE_2_ in HCV replicon cells, which was consistent to our observation (Fig 1). However, they found that HCV replication was enhanced by COX-2 inhibitor treatment [47], which was in contrast to our results (Fig 7B). The opposite results may be due to COX-2 inhibitor treatment at different time course (24 hrs compared with 72 hrs) or the different adaptive mutation of NS5A with different effect on COX-2 regulation in different HCV replicon cell lines. Although there are studies showing that IL-8 can partially influence the antiviral response of interferons to augment viral replication [48], the influence of COX-2 or even PGs expression on HCV replication remains unclear. In present study, we found that IL-8 enhances HCV RNA replication through COX-2 induction and attenuates the replication on treatment with NS398 (Fig 7). These results indicated that IL-8-mediated COX-2 expression is advantageous for HCV replication. Taken together, we identified the mechanism of auto-enhanced replication of HCV through the IL-8/COX-2-dependent pathway.

In summary, we demonstrated that IL-8 levels were significantly elevated by HCV expression or NS5A overexpression. HCV-induced IL-8 production increased the phosphorylation levels of ERK and JNK, which in turn activated the nuclear translocation of C/EBP, promoting COX-2 expression. However, the detailed mechanism by which COX-2 augments viral replication needs to be further elucidated. We provide a novel insight into HCV-induced COX-2 regulation through the IL-8 inflammatory pathway. In addition, we propose a pharmacological approach of targeting IL-8 or COX-2 production, which may provide a potential novel strategy in adjuvant development for anti-HCV therapy.

## Supporting Information

S1 Fig Analysis of the effects of IL-8 on ERK and JNK signaling pathways. (A) Huh7 cells were treated with IL-8 for 2 hr. The cell lysates were collected and subjected to western blotting with specific antibodies for MAPK (ERK and JNK) and phospho-MAPK (ERK and JNK). GAPDH was used an equal loading. (B) The Huh7 cells were treated with IL-8 combined with or without the specific ERK inhibitor U0126 and JNK inhibitor SP600125. At indicated time points, the cell supernatant was analyzed to quantify the amount of IL-8 using an ELISA kit, as described in the Materials and Methods (12 hrs /left panel, 24hrs /right panel). The experiments were performed in three independent. Data shown are mean ± SE; n = 3. *p < 0.05. **p < 0.01.
(TIFF)

S2 Fig Effects of HCV structural proteins on COX-2 promoter activity. Huh7 cells were co-transfected with pCOX-2-Luc and different viral protein expression vectors (Core, E1, and E2). After incubation for 3 days, the cell lysates were subjected to the luciferase activity assay to measure the induction of the COX-2 promoter. The experiments were performed in three independent. Data shown are mean ± SE; n = 3. *p < 0.05. **p < 0.01.
(TIFF)

S3 Fig Analysis of the effects of IL-8 on PGE_2_ production. The Huh7 cells were transfected with or without the C/EBP shRNA and then treated with IL-8 for 3 days. The amount of intracellular PGE_2_ was analyzed by PGE_2_ ELISA kit, as described in the Materials and Methods. The experiments were performed in three independent. Data shown are mean ± SE; n = 3. *p < 0.05. **p < 0.01.
(TIFF)
