# Supplementary figures and images for: HCV NS5A Up-Regulates COX-2 Expression via IL-8-Mediated Activation of the ERK/JNK MAPK Pathway
Source: PLoS One. 2015 Jul 31;10(7):e0133264. doi: 10.1371/journal.pone.0133264 (PMC4521820; doi:10.1371/journal.pone.0133264)

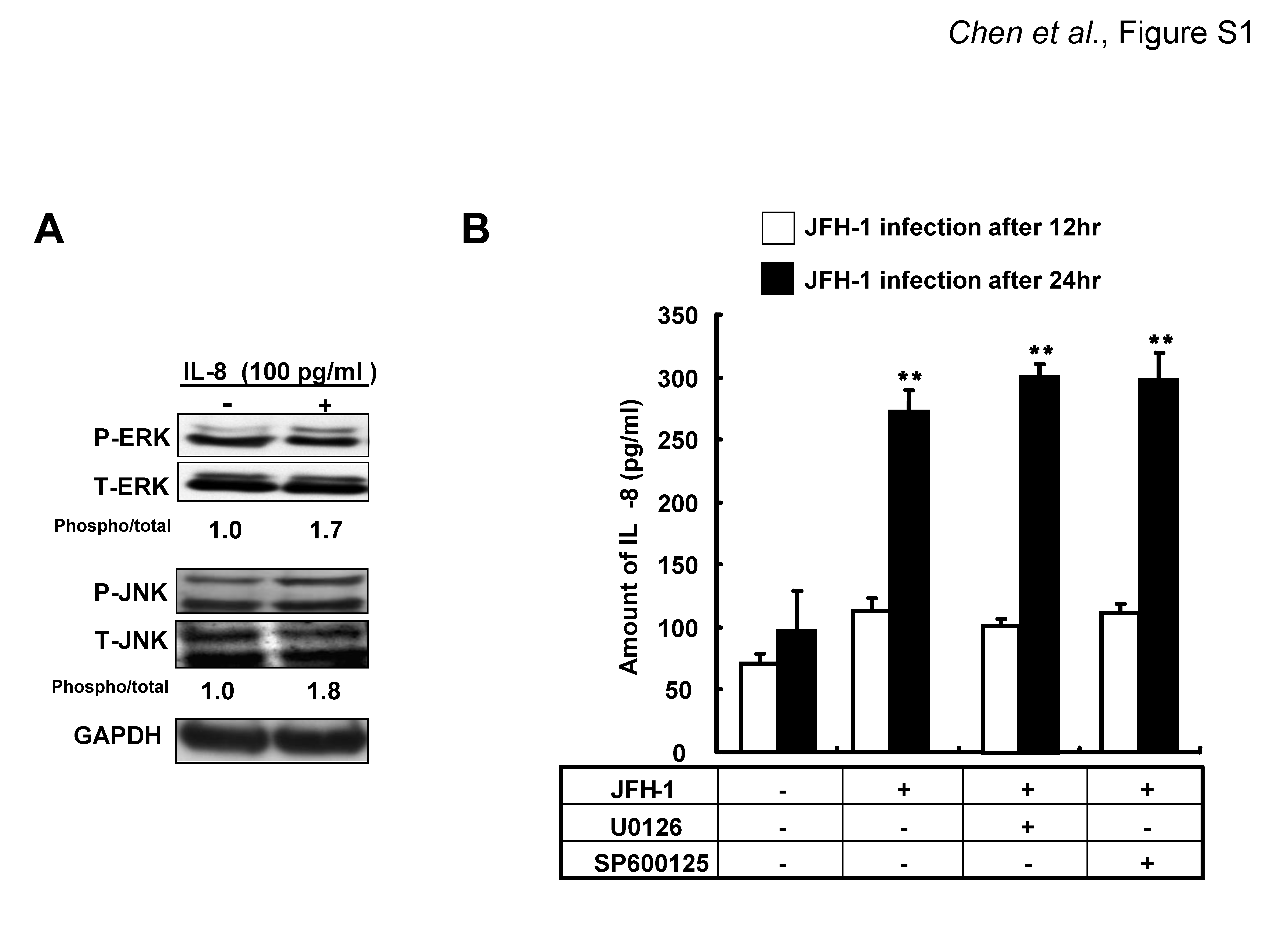

Supplement: S1 Fig — (A) Huh7 cells were treated with IL-8 for 2 hr. The cell lysates were collected and subjected to western blotting with specific antibodies for MAPK (ERK and JNK) and phospho-MAPK (ERK and JNK). GAPDH was used an equal loading. (B) The Huh7 cells were treated with IL-8 combined with or without the specific ERK inhibitor U0126 and JNK inhibitor SP600125. At indicated time points, the cell supernatant was analyzed to quantify the amount of IL-8 using an ELISA kit, as described in the Materials and Methods (12 hrs /left panel, 24hrs /right panel). The experiments were performed in three independent. Data shown are mean ± SE; n = 3. *p < 0.05. **p < 0.01. (TIFF) [file pone.0133264.s001.tiff]

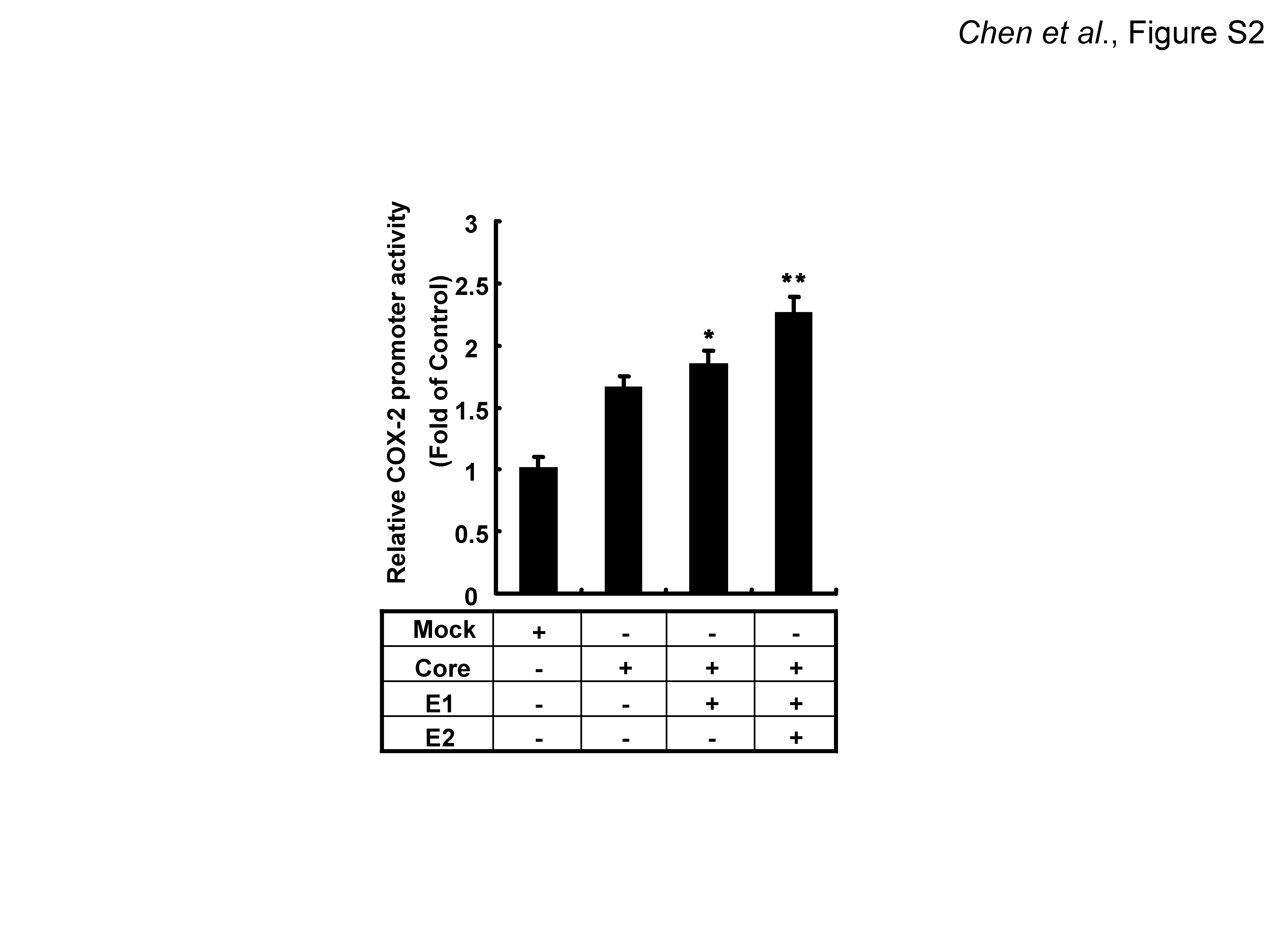

Supplement: S2 Fig — Huh7 cells were co-transfected with pCOX-2-Luc and different viral protein expression vectors (Core, E1, and E2). After incubation for 3 days, the cell lysates were subjected to the luciferase activity assay to measure the induction of the COX-2 promoter. The experiments were performed in three independent. Data shown are mean ± SE; n = 3. *p < 0.05. **p < 0.01. (TIFF) [file pone.0133264.s002.tiff]

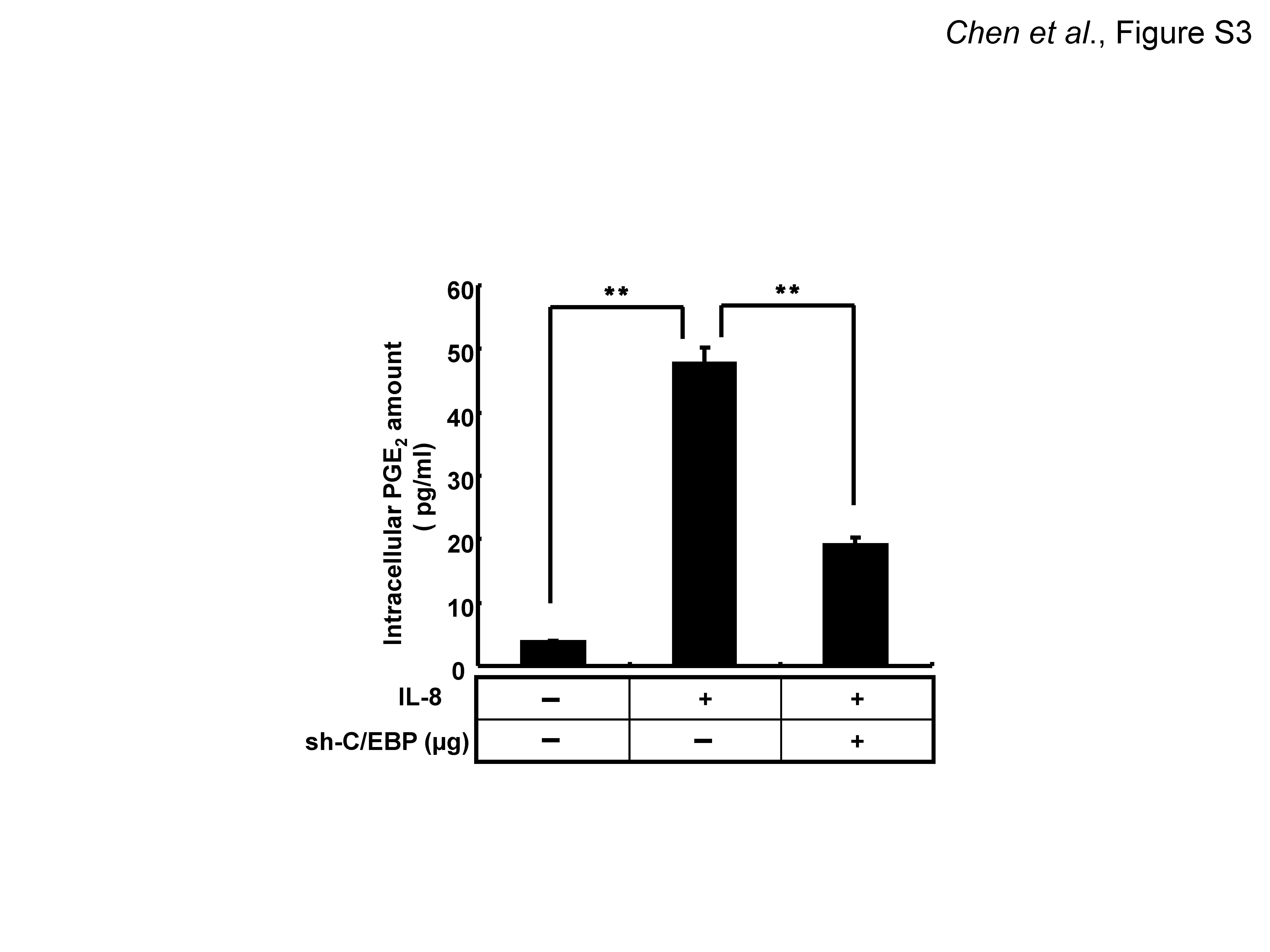

Supplement: S3 Fig — The Huh7 cells were transfected with or without the C/EBP shRNA and then treated with IL-8 for 3 days. The amount of intracellular PGE2 was analyzed by PGE2 ELISA kit, as described in the Materials and Methods. The experiments were performed in three independent. Data shown are mean ± SE; n = 3. *p < 0.05. **p < 0.01. (TIFF) [file pone.0133264.s003.tiff]
